# Harms associated with gambling: abbreviated systematic review protocol

**DOI:** 10.1186/s13643-020-01397-4

**Published:** 2020-06-23

**Authors:** Caryl Beynon, Nicola Pearce-Smith, Rachel Clark

**Affiliations:** 1 Public Health England, Liverpool, UK; 2 Suite 3b, Cunard Building, Water Street, Liverpool, L3 1DS UK; 3 Public Health England, Bristol, UK; 4 Public Health England, London, UK

**Keywords:** Gamble, Harm, Public health, Systematic review, Loot boxes

## Abstract

**Background:**

According to the Gambling Commission, in 2018, almost half of the general population aged 16 and over had participated in gambling in the 4 weeks before being surveyed. Such surveys suggest that the proportion of people who are classed as ‘problem’ gamblers is relatively small; however, this may be related to the ways data are collected and gambling behaviour is classified. Concern about the harms associated with gambling is rising, and in response, Public Health England (PHE) has initiated this review to identify the harms associated with this activity. Harms to the gambler, their close associates and the wider society will all be included.

**Methods:**

Abbreviated systematic review processes will be employed. Ovid MEDLINE, Ovid Embase, Ovid Psycinfo, NICE Evidence and EBSCO SocIndex; a range of websites (for grey literature); and reference lists of included studies will be searched. Experts will be asked to identify other relevant literature. Literature published in years 2005–2019, published in English, from a country within the Organisation for Economic Development (OECD) and following an observational, qualitative or systematic review design will be included. AMSTAR2 (systematic reviews), the Newcastle-Ottawa Scale (observational studies) and the Critical Appraisal Skills Programme (CASP) qualitative checklist (qualitative studies) will be used to assess the risk of bias. A narrative synthesis will be used to summarise the results. The body of evidence will also be assessed according to the principles laid out in the CERQual approach.

**Discussion:**

This protocol provides details of the framework that has been set up to guide this systematic review. The results of this review will provide an extensive assessment of the breadth and magnitude of harms associated with gambling. This will be one of the most comprehensive reviews of gambling-related harms undertaken to date.

**Systematic review registration:**

PROSPERO CRD42019154757

## Background

Gambling is not a new phenomenon, nor is it an uncommon activity. According to the latest survey by the Gambling Commission (the body set up to regulate commercial gambling in Great Britain), in 2018, 46% of the general population aged 16 and over had participated in gambling in the previous 4 weeks, with the National Lottery draw being the most popular form of gambling activity [1].

Concern about the impact of ‘problem’ gambling on society is rising [2]. In 2018, only 0.7% of respondents to the Gambling Commission’s survey were classified as ‘problem gamblers’, with a further 1.1% classified as ‘moderate risk’ gamblers, defined as ‘those who experience a moderate level of problems leading to some negative consequences’ [1]. Data from the same survey showed that, of those that had ever gambled, 6% had self-excluded [1] (i.e. had asked a gambling provider to ban them from gambling for a period of time [3]) indicating that they perceived their own gambling as harmful at some point during their gambling history. Furthermore, 53% of gamblers who were surveyed were not aware of self-exclusion, so the proportion of gamblers who would choose to do this would likely be higher if all were aware this service was available [1].

More importantly, to qualify as a ‘problem’ gambler, a person has to score 8 or more on the Problem Gambling Severity Index (PGSI) or 3 or more according to the Diagnostic or Statistical Manual-IV [4]. This means that the threshold for ‘problem’ gambling is high. A person saying they *sometimes* bet more than they could lose, and whose gambling was *sometimes* causing financial problems, for example, would only score 2 on the PGSI and would therefore be categorised as ‘low risk’, with low risk defined as having ‘few or no identifiable negative consequences’ [1]. In this context, the next category up in terms of frequency that a person can select is ‘most of the time’ so ‘sometimes’ could actually mean frequently. Whether a person who *frequently* bet more than they could lose and whose gambling was *frequently* causing financial problems should be considered at a low risk of harm is questionable. Furthermore, data from surveys like those conducted by the Gambling Commission ask a gambler about their own behaviour and their own perception to the harm they cause, which may or may not capture the full burden to gambling-related harm to others and wider society. Research from Australia using quantitative and qualitative approaches demonstrates that harm occurs at all levels of gambling intensity, not just at ‘problematic’ levels; that harms can persist even once a person has stopped gambling; and that harms are experienced not only by the gambler but by their close associates and the wider community [5].

In general, the minimum legal age for gambling in the UK is 18, although there are exceptions for ‘low stakes’ activities [6]. However, in relation to children, concern has been raised about gambling within the gaming sphere. In some games, micro-transactions enable players to obtain additional content or premiums such as additional powers. Relevant to this discussion are ‘loot boxes’—a reward that is purchased with real money to obtain a random virtual item. Players have to purchase an unknown number of loot boxes before obtaining the item they desire, and the probability of obtaining the item is low. While such practices do not fulfil the legal definition of gambling, they are indistinguishable from activities such as playing slot machines, where the reward is indeterminable, and no skill is required [7]. The Gambling Commission reported that in Great Britain in 2018, 31% of young people aged 11 to 16 who participated in their survey had ever open a loot box in a computer game or app in order to acquire game-related items [8]. The amount of money gamers spend on loot boxes has been linked to problem gambling severity in adults [9].

A preliminary investigation of the literature on the harms associated with gambling shows the broad nature of this issue. Harms include those experienced by the gambler such as suicide [10], some psychiatric disorders (including alcohol dependence [11]) and poor quality sleep [12]; those experienced by close associates such as intimate partner violence [13]; and wider societal harms such as gambling-related crime [14]. In response to growing concerns about these harms, Public Health England (PHE) has initiated this review in order to summarise the body of evidence, assess the quality of the evidence and identify gaps. More specifically, this review will answer the following research questions: (1) what harms are associated with gambling, among children and adults, and (2) what harms are associated with different levels of gambling intensity, among children and adults. A better understanding of the nature, magnitude and breadth of harms is needed before remedial action can be taken.

## Methods

Given the relatively short timescales for this review, an abbreviated methodology will be used which uses recognised techniques to provide a systematic assessment of the evidence in a short timescale compared to a traditional review [15]. The EPPI Reviewer software will be used to manage the records and data throughout the review. Any major deviations from this protocol will be documented by the review team and reported when the review is published.

### Definitions

Gambling: the Gambling Act (2005) defines gambling as ‘any kind of betting, gaming or playing lotteries. Gaming means taking part in games of chance for a prize (where the prize is money or money’s worth), betting involves making a bet on the outcome of sports, races, events or whether or not something is true, whose outcomes may or may not involve elements of skill but whose outcomes are uncertain and lotteries (typically) involve a payment to participate in an event in which prizes are allocated on the basis of chance’ [16].

**Fig. 1:**
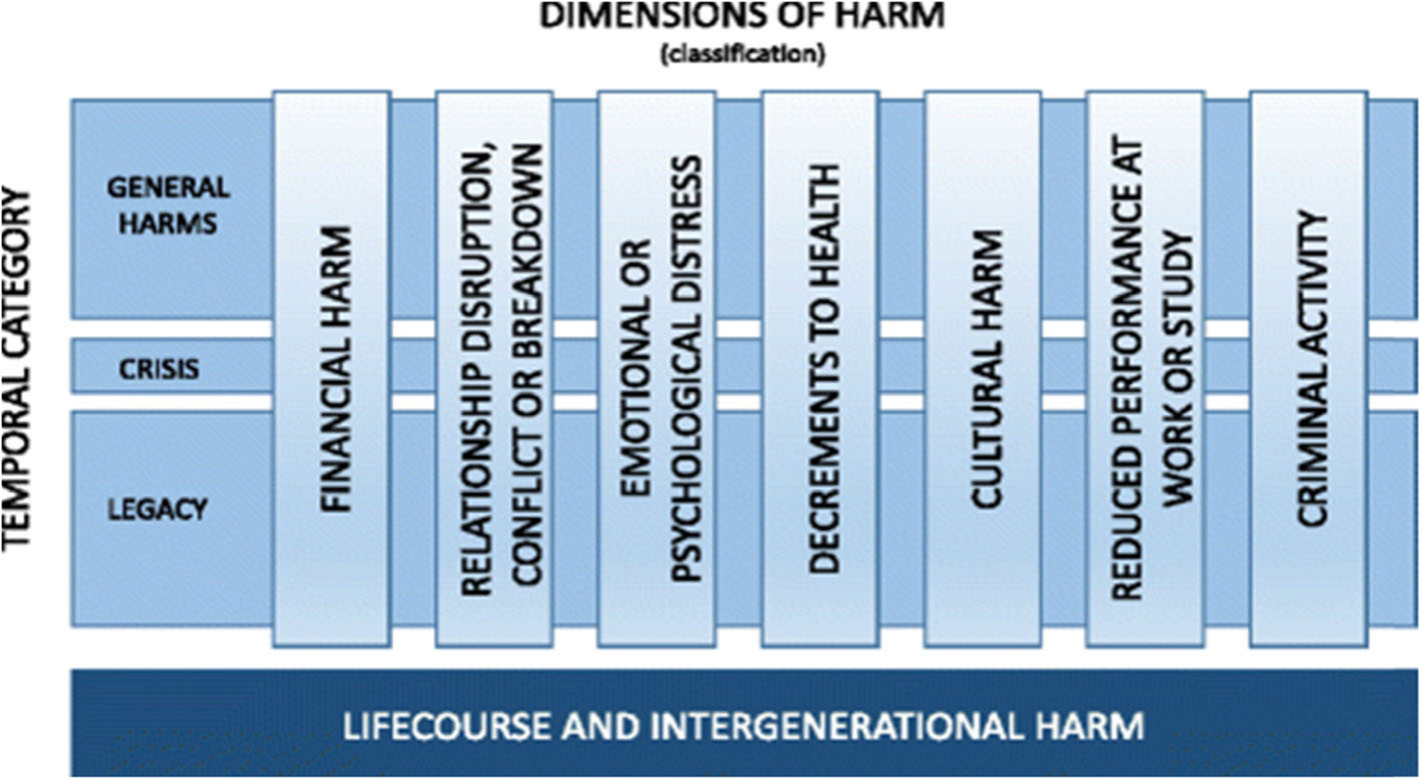
Conceptual framework of gambling-related harm [17]

Gambling-related harm: ‘gambling-related harms are the adverse impacts from gambling on the health and wellbeing of individuals, families, communities and society’ [16]. Scoping work was undertaken to support the development of this protocol. As there was no definitive, internationally agreed definition of gambling-related harm [17], the conceptual framework presented in Fig. 1 is used to understand the different dimensions of harm. Harms to child gamblers include both those related to ‘traditional’ forms of gambling and those related to gambling aspects of gaming.

Gambling intensity: this evidence review aims to identify harms according to different levels of gambling intensity. As such, it aims to identify the harms associated with gambling per se and also the negative consequences associated with gambling at different levels of engagement, for example, low frequency/low spending gambling or gambling at problematic levels. This review will include papers which define problematic gambling in different ways, for example, according to screening tools such as the Diagnostic and Statistical Manual of Mental Disorders IV (DSM-IV) and the Problem Gambling Severity Index (PGSI). The DSM-IV contains 10 diagnostic criteria, and possible scores are between 0 and 10; a score of 3 or over indicates problem gambling. The PGSI contains 9 diagnostic criteria, and a score of between 0 and 27 is possible; a score of 1–2 is ‘low risk’, 3–7 is ‘moderate risk’ and 8 and over is ‘problem gambling’ [4].

### Eligibility criteria

The PICO (population, intervention/issue, comparison and outcome) framework is typically used to develop a review [15] and will be used here (Table 1). Study design has been added in order to better focus the work. Timeframe (follow-up time) is not applicable.

**Table 1:** PICO parameters

	Harm to….		
	Gamblers	Close associates of gamblers (e.g. friends/family)	Wider society
Population	Adults and children, all ages, including studies which focus on sub-groups of the population (e.g. by sex, deprivation, geographical location or from an institution)		
Issue	Gambling (to include all forms of gambling, including gambling-related aspects of gaming, see the ‘Definitions’ section)		
Comparison	None (descriptive studies), any (for comparative studies, e.g. non-gambling general population versus gamblers)		
Outcome	Harms to gamblers (e.g. financial, emotional, health)	Harms to close associates of gamblers (e.g. relationship conflict, child neglect)	Harms to wider society (e.g. crime, work absenteeism)

#### Other inclusion criteria

- Language: English (other languages will not be included, due to the team’s inability to translate)
- Publication date:
  - Stage 1: 2015–2019
  - Stage 2: 2005–2014 (2005 was chosen because in this year the Government issued proposals to reform the law on gambling [i.e. the Gambling Act] and because in 2005/2006 the Economic and Social Research Council/Responsibility in Gambling Trust provided £1 million of funding for research on problem gambling which significantly increased research capacity in this area [18])
- Study design:
  - Stage 1: observational and qualitative
  - Stage 2: systematic reviews of primary studies, including integrative reviews (which combine quantitative and qualitative studies) and meta-analyses
- Publication type: peer reviewed and grey literature
- Setting: studies which are based within the Organisation for Economic Co-operation and Development (OECD). For systematic reviews, some papers may be from countries not within the OECD; this is acceptable if the majority of papers included in the review are. Primary studies which include more than one country may also include non-OECD countries, and inclusion/exclusion will be considered on a case-by-case basis.

#### Exclusion criteria

- Study design:
  - Mapping reviews, scoping reviews, reviews of reviews (‘umbrella’ reviews) and narrative reviews which do not report a formal methodology (‘opinion pieces’)
  - Studies where the primary/secondary aim of the study is not focused on identifying the harms associated with gambling
  - Studies which assess the effect of an intervention

### Search strategy

A comprehensive search will be undertaken using multiple methods to identify both academic and grey literature. The search strategy was developed by a Senior Information Scientist in PHE and peer reviewed by a second Information Specialist in PHE.

#### Electronic searches

The following databases will be searched: Ovid MEDLINE, Ovid Embase, Ovid Psycinfo, NICE Evidence and EBSCO SocIndex. The number of papers retrieved from each database will be recorded. The Ovid MEDLINE search is presented in additional file 1; this will be translated for other databases. The search will look for terms in the title, abstract, author keywords and thesaurus terms (such as MeSH (Medical Subject Headings) in MEDLINE) where available. To search for systematic reviews, a validated review filter will be used for the Ovid databases, and the NICE Evidence searches will be limited to Secondary Evidence only. A validated review filter is currently unavailable for use in EBSCO SocIndex, so a set of search terms will be created in order to search for reviews.

#### Grey literature

Reports and other relevant literature that may not be published in databases will be sought by searching Google and websites such as those listed below. The keywords will be gamble, gambling, betting, casino, lottery, lotteries and loot box. Each website will also be browsed.

- Gamble Aware InfoHub
- Gambling Commission
- GambLib (Gambling Research Library)
- Gam Care
- National Problem Gambling Clinic
- Gordon Moody Association
- Gamblers Anonymous
- Open Grey
- Gam-Anon
- Gambling Information Resource Office Research Library
- Advisory Board for Safer Gambling
- Gambling Watch UK
- Australian Gambling Research Centre
- Gambling Research Exchange Ontario
- Citizens Advice Bureau
- Be Gamble Aware
- Problem Gambling, Wigan Council
- Gambling Compliance
- Gambling Watch UK
- Child Family Community Australia
- International Centre for Youth Gambling Problems and High-Risk Behaviours
- Gambling and Addictions Research Centre
- Alberta Gambling Research Institute
- Responsible Gambling Council
- Problem Gambling Foundation of New Zealand
- Gambling Commission New Zealand
- Victorian Responsible Gambling Foundation

#### Additional searches

Reference lists of included papers will be searched for additional relevant papers which fulfil the inclusion/exclusion criteria. Reviews of reviews identified in stage 2 searches will be obtained; systematic reviews will be extracted and assessed according to the inclusion criteria. After screening, a list of included papers will be shared with the Expert Reference Group (consisting of a number of topic and methodological experts, internal and external to PHE, who will help guide the review). Members will be asked to send any additional papers to the Review Team, and these will be assessed according to the inclusion/exclusion criteria. Any which fulfil the criteria will be included in the review.

### Main and secondary outcomes

Given the breadth of this review, in terms of harms, the types of gambling and different ways gambling intensity is measured, it is not possible to provide specific primary and secondary outcomes. More generally, the primary outcomes are harms associated with gambling and the harms associated with gambling of different intensities.

### Screening

Screening will be undertaken by three reviewers. Pilot work has shown that some references identified in the search for systematic reviews are primary studies. A preliminary title/abstract screen will be undertaken on references identified in the stage 2 search in order to exclude primary studies. The references will be split between the three reviewers, so each will screen a third of the references; 20% of each reviewer’s screened references will be selected and checked for accuracy by a second reviewer. If the agreement is less than 90%, the reason will be explored and rectified and screening will be repeated, in line with the guidance from the National Institute for Health and Care Excellence (NICE) on title/abstract screening [19]. Disagreements will be resolved by the reviewers; a third person (RC) will be consulted if the reviewers cannot come to an agreement.

All remaining references (all from stage 1 and those remaining from stage 2) will be divided between three reviewers. The title/abstract of every reference will be screened independently by two reviewers (‘review pairs’) according to the inclusion/exclusion criteria, and each reference will be coded as either ‘included’ or ‘excluded’. EPPI Reviewer will be used to measure inter-rater agreement for each of the three reviewer pairs; agreement of 90% or over will be considered acceptable. If the agreement is less than 90%, the reason will be explored and rectified and screening will be repeated, in line with the guidance from the National Institute for Health and Care Excellence (NICE) on title/abstract screening [19]. Disagreements will be resolved by the reviewers; a third person (RC) will be consulted if the reviewers cannot come to an agreement.

The full articles of the remaining references will be obtained. Full articles will be divided between reviewers and screened independently using inclusion/exclusion codes set up in advance by the Project Team. Every full-text article will be screened by two people. A third person (RC) will be consulted to resolve disagreements which cannot be resolved by the reviewers. A list of excluded full-text articles and the reason for their exclusion will be reported.

Grey literature will also be screened independently by two reviewers according to the inclusion/exclusion criteria. Disagreements will be resolved between the reviewers, and RC will be consulted if agreement cannot be reached.

### Data extraction

Given the breadth of information, data extraction tables will be used to extract the relevant information from each study. These will include the following information: authors, date, country/setting, the population, the definition of gambling (including how this is measured), comparison (if any and how this is measured), the type of harm, relevant results and funding, plus details of the search for systematic reviews. Authors of included papers will be contacted by the reviewers to ask for missing information or clarification where necessary; this will only occur for information considered essential by the Project Team and a cut-off date will be provided to authors, so that the review process is not delayed. Data extraction tables will be pilot tested before being used and signed off by the Expert Reference Group. All three reviewers will extract the data independently from the eligible studies; all extracted data will be checked by a second reviewer. A third person (RC) will be consulted to resolve disagreements which cannot be resolved by the reviewers.

### Risk of bias assessment

The quality of systematic reviews will be assessed using the AMSTAR2 checklist [20]. The Newcastle-Ottawa Scale will be used to assess the quality of observational studies [21]. The Critical Appraisal Skills Programme qualitative checklist will be used to assess the quality of qualitative studies [22]. Where necessary, these tools will be amended. All papers will be assessed independently by two reviewers. Any discrepancies which cannot be resolved by the reviewers will be resolved by a third person (RC).

### Analysis

The team will undertake a mapping exercise to identify primary papers which are included in more than one systematic review. The implications of this ‘double counting’ will be assessed. For example, a systematic review might be eliminated from the review if all of its primary studies are included in other higher quality reviews.

The heterogeneity of studies included in the review is unlikely to support a quantitative analysis. Instead, a narrative synthesis will be used, with text used to summarise and explain findings [23]. Studies will be summarised according to themes. An appraisal of the quality of the literature will be included. Differences by sub-group (for example by PROGRESS-Plus characteristics [24]) will be provided where this is reported in the literature.

Given the heterogeneity of the evidence included in the review, the body of evidence will be assessed according to the four principles laid out in the CERQual approach which are (1) the methodological limitations of the studies which make up the evidence, (2) the relevance of findings to the review question, (3) the coherence of the findings and (4) the adequacy of the data supporting the findings [25].

## Discussion

This systematic review, following an abbreviated process, will identify the harms associated with gambling. Harms to the gambler, to their close associates and to wider society will be reported. This review is part of a wider piece of work being undertaken by PHE to understand better the public health implications of gambling, in response to growing concerns about such harms. This will be one of the most extensive reviews of harms undertaken to date.

The strengths of this review include the rigorous systematic methods, a detailed search of both published and grey literature and the identification of additional literature from an Expert Reference Group. This review has already been registered with PROSPERO, the register of systematic reviews. Given the wide scope of the review and resulting heterogeneity of studies included, a challenge will be to provide a comprehensive synthesis of the data.

## Supplementary information

**Additional file 1.** The Ovid MEDLINE search.

## Data Availability

Not applicable.

## Abbreviations

AMSTAR: Assessing the Methodological Quality of Systematic Reviews
CASP: Critical Appraisal Skills Programme
CERQual: Confidence in the Evidence from Reviews of Qualitative Research
DSM: Diagnostic and Statistical Manual
NICE: National Institute for Health and Care Excellence
OECD: Organisation for Economic Co-operation and Development
PGSI: Problem Gambling Severity Index
PHE: Public Health England
