# Supplementary material for: Harms associated with gambling: abbreviated systematic review protocol
Source: Syst Rev. 2020 Jun 23;9:148. doi: 10.1186/s13643-020-01397-4 (PMC7313181; doi:10.1186/s13643-020-01397-4)
Supplement: Supplementary file 1 — Additional file 1. The Ovid MEDLINE search. [file 13643_2020_1397_MOESM1_ESM.docx]

**Additional file 1**

The Ovid Medline search is presented here; this will be translated for other databases.

| 1. gambl*.tw,kw. |
| --- |
| 2. (Iowa adj gambl*).tw,kw. |
| 3. 1 not 2 |
| 4. Gambling/ |
| 5. virtual good*.tw,kw. |
| 6. (lottery or lotteries or lotto).tw,kw. |
| 7. (scratchcard* or scratch card*).tw,kw. |
| 8. in-game purchase*.tw,kw. |
| 9. game credit*.tw,kw. |
| 10. (loot box* or loot crate*).tw,kw. |
| 11. slot machine*.tw,kw. |
| 12. fruit machine*.tw,kw. |
| 13. (video lottery or VLT).tw,kw. |
| 14. casino*.tw,kw. |
| 15. amusement arcade*.tw,kw. |
| 16. microtransaction*.tw,kw. |
| 17. (bingo not gene).tw,kw. |
| 18. ((betting or bet or bets) and (horse* or racing or dog*)).tw,kw. |
| 19. (game or games or gaming or gamer).tw,kw. |
| 20. Video Games/ |
| 21. 19 or 20 |
| 22. (money or monetization or monetisation or monetary or reward* or win or wins or winning* or loss or losses or lose).tw,kw. |
| 23. exp Reward/ |
| 24. 22 or 23 |
| 25. 21 and 24 |
| 26. 3 or 4 or 5 or 6 or 7 or 8 or 9 or 10 or 11 or 12 or 13 or 14 or 15 or 16 or 17 or 18 or 25 |
| 27. harm*.tw,kw. |
| 28. risk*.tw,kw. |
| 29. (impact or impacts).tw,kw. |
| 30. risk/ |
| 31. (normalis* or normaliz*).tw,kw. |
| 32. inequalit*.tw,kw. |
| 33. 27 or 28 or 29 or 30 or 31 or 32 |
| 34. debt*.tw,kw. |
| 35. bankrupt*.tw,kw. |
| 36. pawn*.tw,kw. |
| 37. (loan or loans).tw,kw. |
| 38. deprivation.tw,kw. |
| 39. financial loss*.tw,kw. |
| 40. (financial* adj2 difficult*).tw,kw. |
| 41. credit*.tw,kw. |
| 42. poverty.tw,kw. |
| 43. homeless*.tw,kw. |
| 44. housing instabilit*.tw,kw. |
| 45. Bankruptcy/ |
| 46. exp Poverty/ |
| 47. Housing/ |
| 48. communit*.tw,kw. |
| 49. Residence Characteristics/ |
| 50. Socioeconomic Factors/ |
| 51. 34 or 35 or 36 or 37 or 38 or 39 or 40 or 41 or 42 or 43 or 44 or 45 or 46 or 47 or 48 or 49 or 50 |
| 52. irritabil*.tw,kw. |
| 53. mood*.tw,kw. |
| 54. emotional* distress*.tw,kw. |
| 55. psychological* distress*.tw,kw. |
| 56. (anxiety or anxious).tw,kw. |
| 57. (depressed or depression).tw,kw. |
| 58. (substance use* or substance abuse* or substance misuse).tw,kw. |
| 59. shame*.tw,kw. |
| 60. stigma.tw,kw. |
| 61. isolation.tw,kw. |
| 62. (loneliness or lonely).tw,kw. |
| 63. ("quality of life" or resilien*).tw,kw. |
| 64. exp Stress, Psychological/ |
| 65. Irritable Mood/ |
| 66. Anxiety/ |
| 67. Depression/ |
| 68. Substance Related Disorders/ or Drug Users/ |
| 69. Social Stigma/ |
| 70. exp Guilt/ |
| 71. Social Isolation/ |
| 72. Quality of Life/ or Resilience, Psychological/ |
| 73. 52 or 53 or 54 or 55 or 56 or 57 or 58 or 59 or 60 or 61 or 62 or 63 or 64 or 65 or 66 or 67 or 68 or 69 or 70 or 71 or 72 |
| 74. (mortality or death or deaths).tw,kw. |
| 75. morbidity.tw,kw. |
| 76. (co?morbidit* or multi?morbidit*).tw,kw. |
| 77. ill health.tw,kw. |
| 78. insomnia*.tw,kw. |
| 79. hypertension.tw,kw. |
| 80. heart disease*.tw,kw. |
| 81. cardiovascular disease*.tw,kw. |
| 82. stomach problem*.tw,kw. |
| 83. peptic ulcer*.tw,kw. |
| 84. migraine*.tw,kw. |
| 85. neglect.tw,kw. |
| 86. poor nutrition.tw,kw. |
| 87. ((lack adj2 exercis*) or inactivit*).tw,kw. |
| 88. (non-complian* adj2 medic*).tw,kw. |
| 89. self-harm*.tw,kw. |
| 90. suicid*.tw,kw. |
| 91. addiction.tw,kw. |
| 92. Mortality/ |
| 93. Morbidity/ or exp Comorbidity/ |
| 94. "Sleep Initiation and Maintenance Disorders"/ |
| 95. Hypertension/ |
| 96. Heart Diseases/ |
| 97. Cardiovascular Diseases/ |
| 98. Peptic Ulcer/ |
| 99. Migraine Disorders/ |
| 100. Substance Withdrawal Syndrome/ |
| 101. Self-Neglect/ |
| 102. Malnutrition/ |
| 103. Medication Adherence/ |
| 104. Self-Injurious Behavior/ |
| 105. Suicide/ |
| 106. Alcoholism/ |
| 107. 74 or 75 or 76 or 77 or 78 or 79 or 80 or 81 or 82 or 83 or 84 or 85 or 86 or 87 or 88 or 89 or 90 or 91 or 92 or 93 or 94 or 95 or 96 or 97 or 98 or 99 or 100 or 101 or 102 or 103 or 104 or 105 or 106 |
| 108. divorce*.tw,kw. |
| 109. (relationship adj2 break*).tw,kw. |
| 110. (marriage adj2 break*).tw,kw. |
| 111. (relationship adj2 conflict*).tw,kw. |
| 112. (child?care or child care).tw,kw. |
| 113. (parent* or family or families or guardian* or mother or maternal or father or paternal or mum or mom or dad).tw,kw. |
| 114. violen*.tw,kw. |
| 115. Divorce/ |
| 116. Family Relations/ or exp Parents/ or "Child of Impaired Parents"/ |
| 117. Family Conflict/ |
| 118. Child Care/ |
| 119. exp Domestic Violence/ |
| 120. exp Violence/ |
| 121. 108 or 109 or 110 or 111 or 112 or 113 or 114 or 115 or 116 or 117 or 118 or 119 or 120 |
| 122. cultural belief*.tw,kw. |
| 123. cultural practice*.tw,kw. |
| 124. cultural role*.tw,kw. |
| 125. Culture/ |
| 126. 122 or 123 or 124 or 125 |
| 127. (economic activit* or economic inactivit*).tw,kw. |
| 128. absenteeism.tw,kw. |
| 129. productivity.tw,kw. |
| 130. presenteeism.tw,kw. |
| 131. truancy.tw,kw. |
| 132. (school adj2 attainment).tw,kw. |
| 133. (loss adj2 employment).tw,kw. |
| 134. unemploy*.tw,kw. |
| 135. (loss adj2 wage*).tw,kw. |
| 136. Economic Status/ |
| 137. Absenteeism/ |
| 138. Presenteeism/ |
| 139. Academic Failure/ |
| 140. Unemployment/ |
| 141. 127 or 128 or 129 or 130 or 131 or 132 or 133 or 134 or 135 or 136 or 137 or 138 or 139 or 140 |
| 142. (crime or crimes or criminal).tw,kw. |
| 143. fraud*.tw,kw. |
| 144. (child neglect or child maltreatment or parental conflict*).tw,kw. |
| 145. trafficking.tw,kw. |
| 146. prostitution.tw,kw. |
| 147. (theft* or robber* or burglar*).tw,kw. |
| 148. scam*.tw,kw. |
| 149. Crime/ |
| 150. exp Fraud/ |
| 151. Human Trafficking/ |
| 152. Theft/ |
| 153. Child Abuse/ |
| 154. 142 or 143 or 144 or 145 or 146 or 147 or 148 or 149 or 150 or 151 or 152 or 153 |
| 155. 33 or 51 or 73 or 107 or 121 or 126 or 141 or 154 |
| 156. 26 and 155 |
| 157. limit 156 to English language |

Note: BiNGO is a product used in analysing gene experiments so have included a ‘NOT gene’ to exclude this. The Iowa Gambling task was appearing quite regularly which was not relevant, so have excluded this by using ‘NOT Iowa adj gambl*
